# Assessment of preschool preparedness intervention package on adoption of nutrition friendly school initiative in rural Sindh, Pakistan: a pre-and post-intervention design

**DOI:** 10.3389/fnut.2024.1395883

**Published:** 2024-08-01

**Authors:** Amir Ali Samnani, Rozina Nuruddin, Pammla Petrucka, Sajid B. Soofi, Rozina Karmaliani

**Affiliations:** ^1^Department of Community Health Sciences, Aga Khan University, Karachi, Pakistan; ^2^School of Nursing and Midwifery, Aga Khan University, Karachi, Pakistan; ^3^College of Nursing, University of Saskatchewan, Saskatoon, SK, Canada; ^4^Department of Pediatric and Child Health, Aga Khan University, Karachi, Pakistan; ^5^The Brain and Mind Institute, Aga Khan University, Karachi, Pakistan

**Keywords:** early child development, nutrition interventions, malnutrition, nutrition friendly school initiative, preschool children, school community, school preparedness, school nutrition

## Abstract

**Background:**

In 1995, the World Health Organization launched its Global School Health Initiative intending to expand this health promotion approach throughout schools globally. In this study, we aim to assess the preparedness of preschools in the adoption of intervention packages under the Nutrition Friendly School Initiative (NFSI) checklist.

**Methods:**

From three campuses of the Aga Khan School located in the Thatta and Sujawal districts of the Sindh province, Pakistan, all eligible preschool children were selected for this study. Using a pre-and post-intervention design, we assessed preschool preparedness using the NFSI checklist, knowledge of parents/caregivers regarding health and nutrition promoting behaviors, and anthropometric measurements (i.e., mid-upper arm circumference (MUAC), weight, and height) for preschool children. The NFSI checklist was analyzed with differential scores, while descriptive statistics were used for anthropometric and knowledge data. Continuous variables (i.e., height, weight, MUAC) were presented as means, while categorical variables (knowledge) were expressed as numbers and percentages. Paired t-tests for dependent samples were used to statistically assess mean differences in MUAC, height, weight, height-for-age Z-score, weight-for-age Z-score, weight-for-height Z-score, and changes in parental knowledge of preschool children.

**Results:**

Data from 164 preschool children (ages 24–84 months, mean age 56.7 months) were analyzed over 3 months. School preparedness scores improved from 10 to 22 points (out of 26). Parental knowledge on nutrition and health increased by 7.2 points (out of 25). Children showed mean increases in MUAC (0.27 cm), weight (0.36 kg), and height (0.62 cm) (*p* < 0.001). Stunting and overweight/obesity rates remained the same (7.3 and 4.3%), while underweight and wasting rates dropped from 10.4 to 7.3% and 7.9 to 6.1%, respectively. The initiative effectively reduced underweight and wasting but did not impact stunting and overweight.

**Conclusion:**

The NFSI has greatly enhanced preschool readiness for nutrition-friendly schools. Engaging the private sector in addressing nutritional challenges has paved the way for future public-private partnerships to tackle malnutrition. The nutrition policy formulated through this initiative could serve as a blueprint for a National School Nutrition Policy.

## Introduction

An essential component of a child’s journey toward optimal growth and development is ensuring they receive adequate nutrition. Child malnutrition manifests in three broad forms: undernutrition, which includes stunting (low height-for-age), wasting (low weight-for-height), and underweight (low weight-for-age), overnutrition (overweight and obesity), and micronutrient-related malnutrition (1). The United Nations Sustainable Development Goal 2 calls for an end to all forms of malnutrition by 2030 (2).

In the realm of malnutrition, South Asia bears the greatest global burden, with statistics revealing that the region encompasses 52% of stunting cases, 70% of wasting instances, and 48% of overweight occurrences, a phenomenon often referred to as the “South Asian enigma” (3). Over half of malnourished children in South Asia are concentrated in Pakistan, Bangladesh, and India (3).

A scoping review synthesized evidence on malnutrition prevalence in South Asian children aged 5–19 years. Findings from 296 articles revealed varying rates of undernutrition: thinness (1.9–88.8%), wasting (3–48%), underweight (9.5–84.4%), and stunting (3.7–71.7%) (4). Overweight and obesity rates ranged from 0.2 to 73% and 0 to 38%, with upward trends (4).

Pakistan faces a triple burden of childhood malnutrition, with proportions of 40.2, 17.7, and 29.9% experiencing stunting, wasting, or underweight low weight-for-age, respectively, while 9.5% are overweight (5). In comparison to its neighboring country India, Pakistan has higher prevalence of stunting and overweight. In India, the prevalence of stunting among children under five stands at 35%, with corresponding figures for wasting and underweight at 17 and 33%, respectively. Moreover, 2% of children under five in India are categorized as overweight or obese (6). Additionally, a sizable portion of Pakistani children, totaling 53.7%, suffer from anemia, and 28.6% exhibit iron deficiency anemia. Deficiencies in other micronutrients, including zinc (18.6%), Vitamin A (51.5%), and Vitamin D (62.7%), are also prevalent (5). The prevalence of stunting improved from 1965 (48%) to 1994 (36.3%) but worsened from 2001 (41.6%) to 2011 (43.7%). As of 2018, it remains high at 40.2%, with an average annual reduction rate of 0.5%, insufficient to notably reduce stunting in Pakistan. Similarly, wasting has increased steadily since 1997, rising from 8.6% in 1997 to 17.7% in 2018. Sindh province has the highest rates of wasting (23.3%) and underweight nationwide (41.3%) (5).

In addressing malnutrition, the Government of Pakistan (GoP) has demonstrated significant commitment through various initiatives. The establishment of the National Nutrition Forum (NNF) within the Planning Commission serves as a crucial nutrition governance platform, aiming to lead in coordinating nutrition programs, aligning efforts, and formulating nationwide policies. Furthermore, the Federal Government has recently launched the Pakistan Nutrition Initiative (PANI), a comprehensive national program targeting malnutrition in 36 high-impact districts across the country (7). Recommendations have also been made for the development of a national nutrition dashboard to monitor key indicators, emphasizing the importance of Early Childhood Development (ECD) in fostering resilient and proficient individuals through cognitive, social, emotional, and physical development facilitation (7).

Preschool and school children are increasingly affected by malnutrition. Global shifts to energy-dense but nutrient-poor diets and decreased physical activity have led to rising rates of overweight, obesity, and diet-related noncommunicable diseases (NCDs) (8). Many school-age children skip breakfast, eat too few fruits and vegetables, and consume excessive sugary, salty, and fatty snacks. The low consumption of fruits and vegetables in childhood is particularly concerning given that children are more likely to continue with this similar deficit consumption pattern as adults (9).

The school food environment significantly influences nutrition-related behaviors. Effective school-based interventions that are multi-component and encompass whole-school activities, such as modifying school policies, curricula, and the social and physical environment, alongside engaging families and communities (10). In 1995, the World Health Organization (WHO) launched its Global School Health Initiative (GSHI) guided by the 1986 Ottawa Charter of Health Promotion to expand the Health Promoting School (HPS) approach globally (11). It was designed to improve the health of children and the community through schools at all levels (12).

To counteract the rise of chronic, non-communicable diseases, the WHO has advocated for the implementation of school policies and programs that endorse healthy eating habits and physical activity, operating within a comprehensive school policy framework (13). This initiative is further supported by the Nutrition-Friendly Schools Initiative (NFSI), which was introduced by the WHO after expert consultations on childhood obesity convened in Kobe, Japan in 2005 (12).

Given the crucial role of malnutrition in child development and its subsequent impact on national development, there exists a clear rationale for integrating nutrition interventions into preschool settings (nursery and kindergarten). The primary objective of this study was to assess the preparedness of selected preschools to adopt the NFSI intervention package in Thatta and Sujawal districts of Sindh province. Our secondary objectives were to assess: (i) pre and post-intervention knowledge of parents of preschool children regarding the importance of nutrition in the early years of life and health-promoting behavior, and (ii) pre and post-intervention nutrition status of preschool children of age 24–84 months using mid-upper arm circumference (MUAC) (for children 24–59 months only), height, and weight.

**Figure 1:**
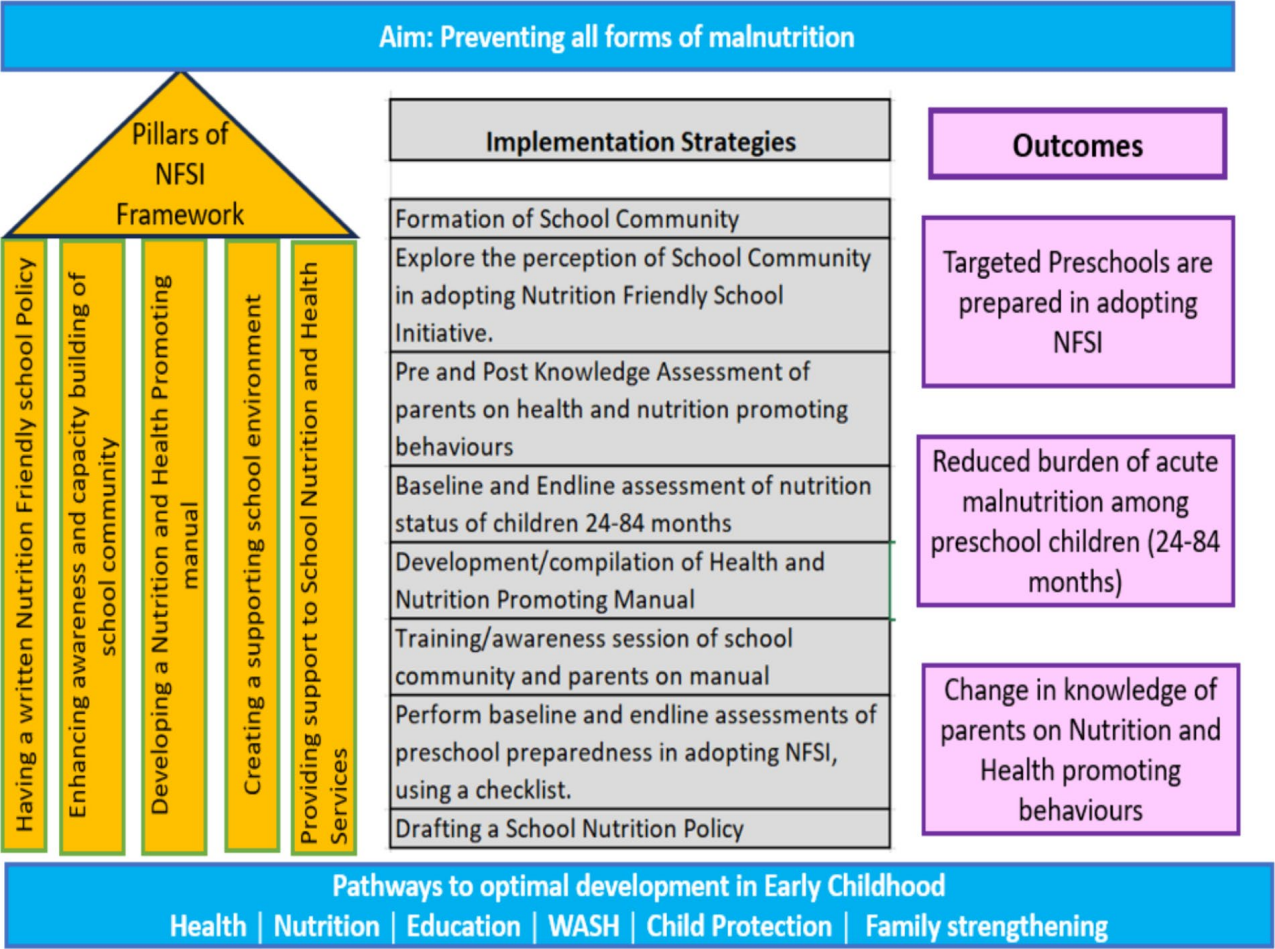
Action framework for implementing nutrition-friendly school initiative. Left side: Five pillars from the framework adopted from the WHO framework for nutrition-friendly school initiative (14). Center: Refined implementation strategies after the initial exploratory phase. Right side: The anticipated outcomes.

The five focus areas (pillars) conceptually outlined in NFSI (Figure 1) provide a framework for self-appraisal (baseline and end line) of preschool readiness. This research has provided an opportunity to establish a nexus between health, nutrition, and education by facilitating early identification through routine screening, establishing referral linkages for those in need, raising awareness among parents and the school community about health-promoting and parenting messages, and sustaining the initiative through school nutrition policies. These efforts aimed to enhance both wellness and long-term developmental trajectories.

## Methods

### Study design

This paper outlines the quantitative component of an exploratory sequential mixed-methods study design that employed a pre- and post-intervention approach. This design enables triangulation and iterative refinement of research questions or intervention strategies (15). In the context of this study, the initial exploratory phase (14) guided the refinement of the intervention package, enhanced understanding of the preschool environment, and assessed the feasibility of adopting and maintaining nutrition-friendly initiatives within available resources.

### Study setting and sites

This study was conducted at three preschool campuses of Aga Khan Education Service, Pakistan (AKES, P) in rural communities in Thatta (Mirpur Sakro and Vur) and Sujawal districts of Sindh province. AKES, P operates 153 schools and five hostels across Pakistan (16). The rationale behind selecting private schools is the feasibility, flexibility, and likely readiness of school management to mobilize resources to implement, adopt, and sustain the intervention as described in NFSI. These schools have a structured pre-primary curriculum run by teachers trained in early childhood development which is not available in current public school’s set-up.

### Study participants

This study involved preschool children aged 24–84 months, along with their parents or direct caregivers. A cross-sectional (complete enumeration) method was employed for anthropometric assessment, encompassing all preschool children attending the selected study sites. To be eligible for participation, children were required to be between 24-84 months of age, maintain at least 50% school attendance in the academic year (excluding new enrollees), exhibit no signs of specified medical complications as outlined in the National Guidelines for Community-based Management of Acute Malnutrition, 2015 (17), and be available at the time of anthropometric assessments.

Likewise, parents or direct caregivers of the same children were considered for training sessions. Eligible participants were those who possessed a basic understanding of Urdu and/or English and expressed willingness to partake in voluntary training sessions. Dropout rates among study participants were evaluated based on preschool child attendance criteria, mandating a minimum attendance of at least 70% during the study period. Notably, no children met the dropout criteria specified above, indicating high adherence to the study protocol.

### Intervention package

The intervention package as outlined in Figure 1 was refined based on suggestions from the exploratory phase (14). It includes establishing a school community (SC), developing a health and nutrition-promoting manual which contains five modules (Early Years Development and its Importance, Malnutrition Consequences, Healthy Eating and Balanced Nutrition, Child Health, Hygiene and Safe Environment, and Parenting), drafting a school nutrition policy and aligned with school handbook under existing school policies (Health and Hygiene, Safety and Security, Parental and Community involvement, Gender Equity, annual school calendar policy, Discipline and Disciplinary Actions and Student Clubs and Societies), conducting awareness-raising sessions for parents (which covers the key messages from the manual), performing anthropometric assessments (height, weight, MUAC), establishing a referral contact point and follow-up for children identified with nutrition needs, and creating a nutrition-themed classroom/corner. All activities align with the NFSI checklist items applicable in the context of the selected school setting. The applicability (of checklist items) was discussed during the inception consultative meeting.

### Data collection and analysis

**Figure 2:**
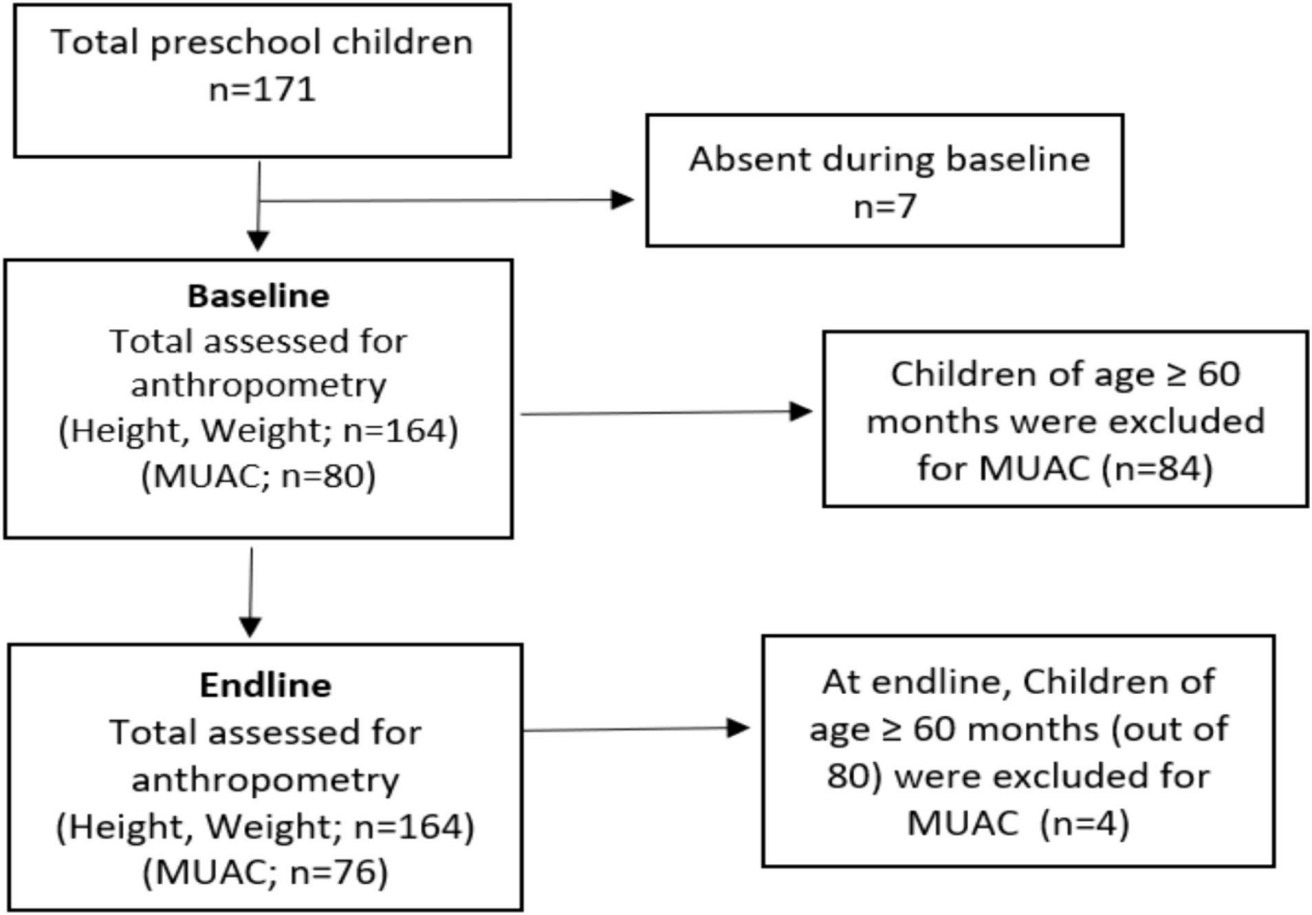
Details of anthropometric assessment for preschool children.

The study spanned a total duration of 6 months, commencing in December 2021 and concluding in May 2022. The interval between baseline and endline anthropometric measurements was 3 months. Quantitative data were collected through the following means:

- *Preschool Preparedness Assessment using NFSI Checklist*: The NFSI checklist, adapted from the WHO report on nutrition actions in school, is comprised of five components and 26 items (13). Each item has three options (Yes, no, and not applicable). Items deemed not applicable were discussed and agreed upon during a consultative workshop with the school community before conducting the baseline assessment. The total score was assigned based on the applicable number of items. This assessment was jointly conducted during a consultative meeting at the time of project closure.
- *Knowledge Assessment of Caregivers:* Knowledge assessment of parents was conducted before and after a four-hour training session using a 25-question true/false based questionnaire. The assessment covered five modules: Early Years Development Importance, Malnutrition Consequences, Healthy Eating and Balanced Nutrition, Child Health, Hygiene and Safe Environment, and Parenting. Parents who consented and attended the training were assessed, and changes in knowledge levels were determined by comparing pre-and post-assessment scores (Supplementary Annexure 2).
- *Anthropometric Assessment*: Pre- and post- anthropometric assessments (MUAC, weight and height) were conducted for preschool children (Figure 2). MUAC among children aged 24–59 months were used to assess wasting in children aged 6–59 months, is a reliable predictor of childhood mortality. The tri-colored MUAC tape categorizes children as normal (green), moderately malnourished (yellow), or severely malnourished (red). The measurement involves marking the midpoint of the left upper arm, between the shoulder and elbow, with the arm bent. The MUAC tape is then wrapped around the arm at the midpoint, and the measurement is taken to the nearest 1 mm.

Preschool children’s weight was measured using a SECA 813 electronic flat scale. Before measurement, shoes, and heavy clothing were removed, and the child stood upright. Measurements were recorded once nearest 0.01 kg. Height measurements were obtained using a wooden height board. Before measurement, shoes and any hair ornaments were removed to prevent interference with readings. Measurements were recorded to the nearest 0.1 cm.

Prior to screening, the school provided student data containing their full name, date of birth and gender, and class details to enable direct data entry onto an Excel spreadsheet and subsequently transferred to Statistical Package for Social Sciences (SPSS™) version 22 for further analysis of anthropometric data obtained at baseline and end-line. Corresponding z-scores were determined to assess stunting, underweight, wasting, and overweight using the WHO AnthroPlus software, which applies the WHO Reference 2007 for 5–19 years to monitor the growth of school-age children and adolescents (18). To ensure continuity with the WHO Child Growth Standards for 0–5 years, these standards are included in AnthroPlus (18).

The primary outcome assessed was the change in preschool preparedness (differential scores) using the NFSI checklist. Secondary outcomes included changes in knowledge among caregivers and the mean changes in MUAC, height, weight, height-for-age Z-scores, weight-for-age Z-scores, weight-for-height Z-scores, or BMI-for-age Z-scores.

Data were analyzed employing both descriptive and inferential statistical techniques. Descriptive statistics were utilized to present continuous variables (i.e., means), while categorical variables were represented as numbers and percentages. For inferential statistics, a Shapiro–Wilk test was conducted to assess the normality of the data obtained from anthropometric measures and knowledge scores. A *p*-value greater than 0.05 indicated that the data were normally distributed. Subsequently, paired *t*-tests were employed to ascertain significant differences in mean anthropometric measures and knowledge scores. The data were stratified by gender and age, and subsequent analyses were conducted to obtain adjusted *p*-values.

### Monitoring and compliance measures

The Co-Principal Investigator (CO-PI) actively participated in the data collection process, training sessions, and consultative workshops. Collaboratively, an activity calendar was developed with schools, and mutually agreed timelines were established. Monitoring of NFSI-specific activities is overseen by the head teacher, utilizing engagement and communication channels to foster accountability. A WhatsApp group was created for the school community to share activity photos and brief reports. As part of this initiative, a school nutrition policy was drafted and integrated into the school handbook, ensuring the systematic inclusion of NFSI-related activities in the school’s annual activity calendar. Consultation meetings at the end line provided opportunities for feedback solicitation and progress assessment, allowing for discussions on areas requiring additional support.

### Ethical consideration

The study protocol was approved by the Ethical Review Committee (ERC) of Aga Khan University—AKU Ref #2021–6,622-20068 (Supplementary Annexure 3). In addition, signed informed consent was obtained from study participants (parents/caregivers), providing clear and concise details about the study purpose, risks, and benefits of participation (Supplementary Annexure 4).

## Results

### Characteristics of children

Among the 164 eligible children, 92 (56.1%) were boys, and, 80 children (48.8%) were aged below 60 months. During the endline assessment, 76 out of 80 were eligible for MUAC, as four children exceeded the age eligibility criteria for MUAC measurement.

### Assessment of pre-school preparedness for NFSI

During the baseline consultation with the school community, only 10 (*n* = 10) out of 26 indicators were marked as “Yes” (score of 10 points). Four indicators were deemed not applicable in the context of the school setting. In the end-line assessment, a total of 22 (*n* = 22) indicators were marked as “Yes” which shows that almost all the applicable areas were addressed through this intervention and the end-line assessment indicates an improvement in school preparedness toward NFSI (Table 1).

**Table 1:** Pre-and-post assessment of preschool readiness based on activities outlined under the NFSI checklist.

		Baseline			End line			Comments
Code	Essential criteria	Yes	No	N/A	Yes	No	N/A	
1	Having a written nutrition-friendly school policy							
1.1	Development of a written Nutrition-friendly Schools’ Policy that addresses all five points described in this framework, and includes elements described in 1.2–1.5		X		X			A draft policy was developed and submitted to the Head of Programs and presented to the AKDN sister organization for review and officiation
1.2	Rationale		X		X			
1.3	Objectives: with timelines and clear milestones		X		X			
1.4	Action plan: Outline the whole-school approach contributing to healthy living.		X		X			The proposed plan has been submitted to the school (embedded with a draft policy document).
1.5	Monitoring and evaluation plan for the Nutrition-Friendly Schools’ Policy.		X		X			Embedded in the draft policy document
2	Enhancing awareness and capacity building of the school community							
2.1	Dissemination of the Nutrition-friendly Schools’ Policy			X			X	Not applicable. The policy document was handed over and discussed with the core team based in Islamabad. Endorsement, dissemination, and adoption will be as per AKESP policy
2.2	Activities for families and community, community involvement, and outreach in the area of nutrition and health-related issues.		X		X			Activities include awareness sessions (training) for parents on thematic areas outlined in Health & Nutrition promoting behavior, periodic health assessment of children, creating referral linkages, counseling of parents identified with malnutrition, engaging children utilizing Nutrition theme classroom, Nutrition month celebrated, and parents were engaged on weekly message (WhatsApp group message). daily WhatsApp message to caregivers on parenting messages (key family care practices). Activities proposed to be aligned with an action plan that mainly includes awareness sessions for parents on thematic areas outlined in Health & Nutrition promoting behavior, periodic health assessment of children, and creating referral linkages.
2.3	School staff training in nutrition and health-related issues.		X		X			School community were trained in nutrition and health-related issues.
3	Developing a nutrition and health-promoting school curriculum							
3.1	Culturally appropriate and effective nutrition education.		X		X			
3.2	Age, sex, and culturally appropriate physical education curriculum.	X			X			
3.3	Healthy Living and life-skills Education Curriculum	X			X			
3.4	Regular monitoring of school curriculum relevant to NFSI, and evaluation of the impact of how well the education meets the objectives.			X			X	Not applicable, beyond the scope of school SoP/policy
4	Creating a supportive school environment							
4.1	School meals, food vendors, and snack bars (if present) promote healthy eating.			X			X	Not applicable, beyond the scope of school SoP/policy.
4.2	Positive messaging toward nutrition and active living.		X		X			Day-wise (weekdays) rolling out of 22 key family care practices (parenting package) in April 2022 Rolled out weekly Health Diet and Health Eating messages on account of National Nutrition Month (in March 2022). Copies of essential material (NNS-2018), Dietary needs of children with 0–8 years, and parenting package have been provided to the school and placed over the Nutrition Theme classroom continuing to roll out positive messages.
4.3	Absence of marketing of foods and beverages at school	X			X			
4.4	Access to an adequate eating place if the school provides food and/or beverages to the children	X			X			
4.5	Adequate school cooking facilities			X			X	Not applicable, beyond the scope of school SoP/ policy.
4.6	Access to safe drinking water.	X			X			
4.7	Promotion of safe hygiene and sanitary behavior.	X			X			Safe hygiene and sanitary behaviors are already practiced however through NFSI these areas were further reinforced through sessions with parents and the school community.
4.8	Availability of clean and separate toilets, for boys and girls.	X			X			
4.9	Opportunity for all age groups to access space and school sporting facilities for physical activity within and outside of the curriculum.	X			X			
4.10	Affirmative action against bullying, stigmatization, and discrimination.	X			X			
4.11	School staff as role models in encouraging healthy eating and healthy lifestyles.	X			X			
5	Providing supportive school nutrition and health services							
5.1	Regular monitoring of children’s growth development.		X		X			Incorporated into a policy document, the school community has received orientation on the growth monitoring of children, focusing on Height, Weight, and Mid-Upper Arm Circumference (MUAC). Additionally, Aga Khan Health Services, Pakistan (AKHS, P) health staff actively participate as volunteers, extending their support for the periodic monitoring, which is conducted semi-annually. Essential supplies, including MUAC tapes, weight machines, and height tapes, have been distributed to each campus. The intervention period saw the implementation of quarterly screenings, a total of two rounds, to comprehensively assess the growth and health of the children.
5.2	The effective feedback system for parents and children on findings of the regular monitoring.		X		X			Incorporated in a policy document, moreover, referral contacts with AKHS, P has been established and AKHS, P staff nurse were also involved in the anthropometric assessment.
5.3	Supportive school health service, including a referral system.		X		X			Linkages with Aga Khan Health Service, Pakistan (AKHS, P) health facilities have been developed and in addition to the school community, AKSH, P staff were engaged as volunteers to support the assessment component. AKHS, P facility will serve as a referral point to ensure a continuum of care.
Total score		10	12	04	22	00	04	

WHO/UNICEF/FAO. (2021). Nutrition-Friendly School Initiative. Part I: Conceptual framework. Part II: Self-appraisal tool. Geneva, Switzerland: World Health Organization (13).

### Pre-post assessment of knowledge among parents of preschool children

The training program focused on key aspects of the health and nutrition-promoting curriculum tailored for parents and caregivers of preschool children. Of the 164 eligible parents invited, 84 individuals (51.2%) participated in the one-day session. Four participants did not submit pre- or post-test and were excluded from the analysis,

At baseline, the mean test score was 12.6 ± 4.92 (50.4%) significantly improving to 19.8/25 ± 3.03 (79.1%) at endline *p* value <0.001 (refer to Table 2). This reflects a substantial increase of 7.2 points in the knowledge of parents/caregivers on health and nutrition promoting manual post-training. Further analysis reveals a greater mean score increase among caregivers of male children (8.3 points) compared to female caregivers (5.2 points). However, the mean difference is negligible across age groups. The overall mean change in knowledge score and changes across age and gender groups are statistically significant (*p* < 0.001) (Table 2).

**Table 2:** Knowledge score of parent/caregiver at baseline and end-line.

Indicators	Mean value/score	Mean difference	*p*-value (95% CI)
Overall knowledge scores in points (out of 25) of caregivers			
Pretest	12.6	7.2	*p* < 0.001 (95% CI: 6.19, 8.15)
Post-test	19.8		
Knowledge scores among children aged under 5 years			
Pretest	11.5	8.4	*p* < 0.001 (95% CI: 5.47, 9.63)
Post-test	19.1		
Knowledge scores among caregivers of children aged 5 years and above			
Pretest	11.0	8.1	*p* < 0.001 (95% CI: 6.41, 9.94)
Post-test	19.1		
Knowledge scores among caregivers of female children			
Pretest	14.8	5.2	*p* < 0.001 (95% CI: 3.36, 6.91)
Post-test	20.0		
Knowledge scores among caregivers of male children			
Pretest	12.4	8.3	*p* < 0.001 (95% CI: 5.27, 11.2)
Post-test	20.7		

### Pre-post anthropometric assessment of preschool children

Anthropometric findings show increase variations in MUAC, weight, and height, with mean increase of 0.27 cm, 0.36 kg, and 0.62 cm, respectively. Among 24–-month-olds participants, boys had a slightly higher mean MUAC increase (0.28 cm) than girls (0.25 cm). Similarly, weight difference was greater in children aged 5 years and above (0.43 kg) than those under 5 years (0.28 kg). However, boys had slightly higher weight differences than girls (0.39 kg vs. 0.33 kg). Overall, mean MUAC changes across genders were statistically significant (*p* < 0.001).

Height differences were negligible between genders and age groups. The mean change in height-for-age Z-score (HAZ) is 0.22, indicating height improvement. HAZ changes were higher in <5-year-olds compared to ≥5-year-olds, with slightly higher differences in girls (0.63) than boys (0.61). Changes were statistically significant across age groups and genders (*p* < 0.001).

Weight-for-age Z-scores (WAZ) also showed a statistically significant mean change (0.06), with overall statistical significance (*p* = 0.002), particularly in <5-year-olds and girls (*p* < 0.01 and 0.013, respectively), but not in ≥5-year-olds (*p* > 0.05).

Weight-for-height Z-score (WHZ) or Body Mass Index-for-Age Z-score (BAZ) had a mean change of 0.11, higher in ≥5-year-olds and boys (0.14). Changes were statistically significant in these groups (*p*-value ≤0.01), but not in <5-year-olds and girls (*p* > 0.05) (Table 3).

**Table 3:** Anthropometric measurements of children at baseline and end-line.

Variables	Overall		Age adjusted				Gender adjusted			
			Under 5 years		Above 5 years		Boys		Girls	
	Difference in mean (95% CI)	*P*-value	Difference in mean (95% CI)	*P*-value	Difference in mean (95% CI)	*P*-value	Difference in mean (95% CI)	*P*-value	Difference in mean (95% CI)	*P*-value
MUAC (cm) for Children 24–59 m	0.27 (0.22, 0.31)	<0.001	0.27 (0.22, 0.31)	<0.001	n/a	n/a	0.28 (0.23, 0.33)	<0.001	0.25 (0.18, 0.31)	<0.001
Weight (Kg) for Children 24–84 m	0.36 (0.28, 0.45)	<0.001	0.28 (0.17, 0.4)	<0.001	0.43 (0.31, 0.56)	<0.001	0.39 (0.27, 0.51)	<0.001	0.33 (0.20, 0.45)	<0.001
Height (cm) for children 24–84 m	0.62 (0.57, 0.68)	<0.001	0.62 (0.54, 0.7)	<0.001	0.63 (0.55, 0.71)	<0.001	0.61 (0.53, 0.68)	<0.001	0.63 (0.55, 0.71)	<0.001
Height-for-age z-score (HAZ)	0.22 (0.2, 0.23)	<0.001	0.27 (0.24, 0.29)	<0.001	0.17 (0.15, 0.19)	<0.001	0.21 (0.19, 0.23)	<0.001	0.22 (0.19, 0.25)	<0.001
Weight-for-age z-score (WAZ)	0.06 (0.02, 0.10)	0.002	0.11 (0.05, 0.17)	<0.001	0.02 (−0.03, 0.03)	0.449	0.04 (0.001, 0.09)	0.053	0.08 (0.02, 0.14)	0.013
Weight-for-Height z-score (WHZ/BHZ)	0.11 (0.05, 0.13)	<0.001	0.008 (0.001, 0.17)	0.052	0.14 (0.06, 0.22)	<0.001	0.14 (0.06, 0.22)	<0.001	0.08 (0.008, 0.16)	0.076

The prevalence of stunting, underweight, wasting, and overweight/obesity was 7.3, 10.4, 7.9, and 4.3%, respectively (Table 4). At the end-line assessment, stunting and overweight/obesity prevalence remained unchanged. However, reductions in underweight (from 10.4 to 7.3%) and wasting (from 7.9 to 6.1%) were observed.

**Table 4:** Change in HAZ, WAZ, and WHZ among children at baseline and end line.

Indicators	Stunted (%)		Underweight (%)		Wasting (%)		Overweight/Obesity (%)	
	HAZ-score ≤ -2SD		WAZ-score ≤ -2SD		WHZ-score ≤ -2SD		WHZ-score > +2SD	
	Baseline	End line	Baseline	End line	Baseline	End line	Baseline	End line
Total malnourished identified (*n* = 164)	12(7.3%)	12(7.3%)	17(10.4%)	12(7.3%)	13(7.9%)	10(6.1%)	7(4.3%)	7(4. 3%)
Child (<5 years of age) (*n* = 77)	1(1.3%)	1(1.3%)	4(5,2%)	1(1.3%)	4(5.2%)	4(5.2%)	3(3.9%)	4(3.9%)
Child (> 5 years of age) (*n* = 87)	11(12.6%)	11(12.6%)	13(14.9%)	11(12.6%)	9(10.3%)	6(9.9%)	4(4.6%)	3(3.4%)
# of boys (*n* = 92)	7(7.6%)	7(7.6%)	9(7.6%)	7(7.6%)	10(10.9%)	8(8.7%)	5(5.4%)	4(4.3%)
# of girls (*n* = 72)	5(6.9%)	5(12.5%)	8(13.9%)	5(6.9%)	3(4.2%)	2(2.8%)	2(2.8%)	3(4.1%)
Moderate(z-score ≤ -2SD and > -3SD) or	11(6.7%)	11(6.7%)	16(10.4%)	12(7.3%)	12(7.3%)	10(6.1%)	4(2.4%)	4(2.4%)
(z-score > +2SD and < +3SD)								
Severe(z-score < -3SD)									1(0.6%)	1(0.6%)	1(1.2%)	0	1(0.6%)	0	3(1.8%)	3(1.8%)

## Discussion

This study assessed the preparedness of selected rural preschools in Sindh for adopting the NFSI to prevent all forms of malnutrition among preschool children by creating a nutrition-friendly preschool environment. The study findings demonstrated progress in transforming selected preschool sites into nutrition-friendly settings, with preparedness scores improving from 10 to 22 points. This improvement resulted from collaborative efforts with school management, including developing a nutrition policy, creating a health and nutrition manual, establishing nutrition screening mechanisms, implementing learn-with-fun approaches through nutrition theme corners, training sessions for mothers, engaging parents in awareness campaigns, and orienting the school community.

The UNICEF-supported NFSI program in Gaza and the West Bank aimed to establish healthy dietary and physical activity habits and improve the nutritional status of school-age children. Implemented through local NGOs, the program supported the Ministry of Education and the Ministry of Health in promoting nutrition and healthy lifestyles. The intervention package elevated school nutrition to a national priority and increased the commitment of Palestinian authorities to future scalable interventions (19).

Anthropometric analysis indicated a higher prevalence of underweight and wasting compared to stunting, with children aged 5 years and above exhibiting a greater burden across all categories of malnutrition (except overweight/obesity). Boys exhibited a higher prevalence than girls in stunting, underweight, wasting, and overweight. The intervention notably reduced underweight and wasting burdens by 29 and 23%, respectively. A similar pattern of malnutrition was observed in a cross-sectional study of school children aged 6–15 years in rural Bengaluru, South India (20), emphasizing the need for targeted interventions to alleviate malnutrition burdens among preschool-age children. Hence, NFSI shows promise in mitigating and preventing various forms of malnutrition.

This study showed a 28.7% improvement in parental knowledge after a training session. From 50.4 to 79.1%. This highlights the role of schools in educating parents about health and nutrition, promoting healthier childhoods. A similar study in Taguig City, Philippines, reported a 22.2% increase in maternal knowledge of nutritious meal preparation (21). Similarly, in Pakistan, a nutrition education intervention in Tando Jam and Quetta for children aged 6 months to 8 years showed significant improvements. In Tando Jam, 36% of children, and in Quetta, 32% of children, improved to normal nutritional status. Meal frequency increased significantly (Tando Jam: *p* ≤ 0.001, Quetta: *p* ≤ 0.025), along with the intake of high-starch foods, vegetables, and fruits in Tando Jam (*p* ≤ 0.001). These results underscore the impact of nutrition education on children’s health and nutrition (22).

Through this initiative, three additional outputs were achieved: (i) drafting of the school nutrition policy; (ii) updating of the health and nutrition promoting behavior manual; and (iii) establishment of nutrition-themed classrooms across all three campuses. Ensuring the sustainability of such initiatives is crucial. The school nutrition policy will serve as a roadmap, aiding in the planning of annual calendars by incorporating activities outlined in the policy. Adequate budget allocation is essential to ensure the quality and effectiveness of these initiatives. Furthermore, the school nutrition manual provides an opportunity to address and prevent health issues by educating parents about nutritional needs and healthy behaviors. The inclusion of a parenting component enhances the manual’s value.

This initiative not only benefited parents/caregivers in improving the knowledge of health and nutrition promoting behavior but also benefited schools in building an enabling environment for promoting the overall health and nutritional well-being of children. It also strengthened the capacity of schools to address the health and nutritional problems of the children, their families, and communities, acting both within and beyond the classroom. Finally, it enabled schools to become accredited as “Nutrition-Friendly Schools,” enhancing the schools’ reputations for making an effective investment in the future generation.

The observed improvement in outcome indicators is attributed to the essential nature of interventions in rural settings. These improvements are further facilitated by the adaptability of school leadership in allocating resources for innovative interventions and strict adherence to monitoring and compliance measures. Additionally, the school’s capabilities, particularly in delivering a well-defined pre-primary curriculum by teachers trained in early childhood development, may have played a significant role. Moreover, increased parental involvement has raised awareness, resulting in improved childcare practices.

Future NFSI implementation in LMICs should extend beyond public sector school settings to include school-age and adolescent children (5–19 years). The intervention should not solely focus on creating an enabling environment but should also integrate school meal and supplementation programs through multi-sectoral collaboration. This endeavor requires sustainable funding, robust monitoring and evaluation, and integration with complementary programs. It must address social determinants such as poverty and gender inequality. Community engagement should inform culturally appropriate interventions encompassing nutrition education, healthy food environments, WASH facilities, and whole-school policies. Coordinated efforts across education, health, agriculture, and local governments are crucial for creating an enabling environment that promotes good nutrition and overall child development.

### Study limitations and strengths

Study limitations include the lack of a control group for this study; therefore, it may limit the internal validity of the study. Without a control group, it is challenging to rule out alternative explanations for observed changes, confounders, or biases (i.e., gender, age predominately). However, to overcome this limitation, internal validity was strengthened when techniques of varying strength were used instead of randomization, including pre- and post-assessment of outcomes. Secondly, the study setting includes private schools only, and therefore the public schools might have different outcomes. Among the strengths, this study used mixed methods research, combining quantitative and qualitative data to optimize the strengths and mitigate the weaknesses of the standalone approach. Notably, this study is pioneering, being the first of its kind to explore NFSI implementation feasibility in rural Sindh. Lastly, it has generated a school nutrition policy, serving as a foundational document to inform national school nutrition policy.

## Conclusion

The NFSI implementation has tremendously improved preschool preparedness toward achieving nutrition-friendly schools. Furthermore, this initiative has significantly improved the knowledge of parents/caregivers on health and nutrition promoting behaviors. This study has engaged the private sector in addressing the nutrition burden and promoting healthy nutrition subsequently opening avenues for future public-private partnerships in alleviating the triple burden of malnutrition.

This study holds the potential for replication in public schools and could pave the way for a school accreditation program, certifying schools as Nutrition-friendly. This move aims to set minimum quality standards for implementing NFSI, as an imperative for holistic child development from an early age. The nutrition policy developed under this initiative has the potential to scale up and inform the development of a National School Nutrition Policy. Future studies in diverse settings, encompassing rural and urban areas as well as private and public schools, are warranted to comprehensively assess the real impact of this intervention.

## Data availability statement

The raw data supporting the conclusions of this article will be made available by the authors, without undue reservation.

## Ethics statement

The studies involving humans were approved by the Aga Khan University, Ethics Review Committee. The studies were conducted in accordance with the local legislation and institutional requirements. Written informed consent for participation in this study was provided by the participants’ legal guardians/next of kin.

## Author contributions

AS: Conceptualization, Data curation, Formal analysis, Investigation, Methodology, Visualization, Writing – original draft, Writing – review & editing. RN: Writing – review & editing. PP: Writing – review & editing, Methodology. SS: Writing – review & editing. RK: Writing – review & editing, Funding acquisition, Project administration, Resources, Supervision.
