# Supplementary material for: Assessment of preschool preparedness intervention package on adoption of nutrition friendly school initiative in rural Sindh, Pakistan: a pre-and post-intervention design
Source: Front Nutr. 2024 Aug 1;11:1395883. doi: 10.3389/fnut.2024.1395883 (PMC11325454; doi:10.3389/fnut.2024.1395883)
Supplement: Supplementary file 1 [file Table_1.docx]

**Annexures**

Annexure 1: Operational definitions/explanations of key terms

| Keywords | Definition/Explanation |
| --- | --- |
| Early child development | ECD encompasses the physical, socio-emotional, cognitive, and motor development of children between 0-8 years of age. |
| Nutrition Interventions | Nutrition interventions are policies, services, learning experiences, and other actions implemented by schools, individuals, or groups to make healthy nutrition a way of daily life |
| Malnutrition | Malnutrition includes overnutrition and nutritional deficiencies as well as undernutrition which impairs health, intellectual activity, adaptive behavior, education, productivity, and well-being, and can induce death. |
| School Community | The school community comprised preschool teachers, a preschool principal, a representative from the preschool management committee, and a parent-teacher committee |
| Nutrition Friendly School Initiative [8] | NFSI includes five components, which represent the conditions or criteria for schools to be considered nutrition-friendly:   - A written school policy on nutrition (also named Nutrition-Friendly Schools’ policy) - Awareness and capacity strengthening of the school community. - Curriculum development and modification. - Creation of a supportive school environment for optimal nutrition and health; - School nutrition and health services |
| Preschool | A preschool, also known as a nursery school, pre-primary school, or play school, is an educational establishment or learning space offering early childhood education to children before they begin compulsory education at primary school. |
| Preschool preparedness | Preschool preparedness refers to a preschool's ability to create a supportive environment that promotes the health and well-being of students by adopting the activities outlined in the Nutrition-friendly school checklist. In the context of this study, preparedness quantitatively indicates achieving either a minimum improvement of 8-10 points or an overall 16-18 points at least at the end line when compared to the baseline. |

**Annexure 2:** **Knowledge Assessment Questionnaire for Parents (Pre-Post Test)**

**Name of Parent: _______________ Name of Child __________________**

Please tick mark your responses

| **S. No** | **Questions** | **True** | **False** |
| --- | --- | --- | --- |
|  | Module 01: Understanding Early years development and its importance | | |
| 1.1 | The first 1000 days of life started from Birth till three years of age. |  |  |
| 1.2 | Adolescence (Age of 10-19 years) is a period of rapid physical, cognitive, social, emotional, and language development. |  |  |
| 1.3 | The brain develops more rapidly when the child first enters school than at any other age. |  |  |
| Module 02: Malnutrition and its consequences | | | |
| 2.1 | Malnutrition refers to undernutrition. |  |  |
| 2.2 | Folic acid deficiency will cause blindness among children |  |  |
| 2.3 | Only doctors and nurses can recognize the signs of malnutrition in children |  |  |
| 2.4 | Undernutrition cannot be prevented |  |  |
|  | Module 03: Healthy Eating and Balanced Nutrition | | |
| 3.1 | Carbohydrates and fats are body-building foods. |  |  |
| 3.2 | Fruits and vegetables are macro-nutrients. |  |  |
| 3.3 | A balanced diet means eating enough quantity of healthy foods. |  |  |
| 3.4 | A child's lunch box is more important than having breakfast |  |  |
| 3.5 | Green leafy vegetables are a good source of Vitamin C |  |  |
|  | Module 04: Child Health, Hygiene, and Safe Environment | | |
| 4.1 | Health is defined as the absence of disease only |  |  |
| 4.2 | A child cannot eat anything when a child has diarrhea |  |  |
| 4.3 | Measles, TB, hepatitis B, and pneumonia cannot be prevented among children. |  |  |
| 4.4 | Vaccinated children can drink tap water without boiling it. |  |  |
| 4.5 | Fruits, vegetables, and meat do not require washing with tap water if to be stored in the refrigerator. |  |  |
| 4.6 | Hands can only be washed when they look visibly dirty. |  |  |
| 4.7 | A safe environment is an environment where children are secure from germs and infectious agents. |  |  |
| 4.8 | Children should not be allowed to play as it may cause injuries and accidents. |  |  |
| 4.9 | If a child is misbehaving at school the teacher can physically punish the child to teach him/her discipline. |  |  |
|  | Module 5: Parenting | | |
| 5.1 | Caring for the child is the responsibility of mothers only |  |  |
| 5.2 | Comforting the child by carrying him/her on lap and telling how much you love him/her is spoiling the child. |  |  |
| 5.3 | Play is only for children; therefore parents should not waste time playing with children because the child will begin to play when he/she is old enough to play with other children. |  |  |
| 5.4 | Brain development is only dependent on good nutrition and the environment does not affect it |  |  |

**Annexure 3: ERC Approval letter**

**
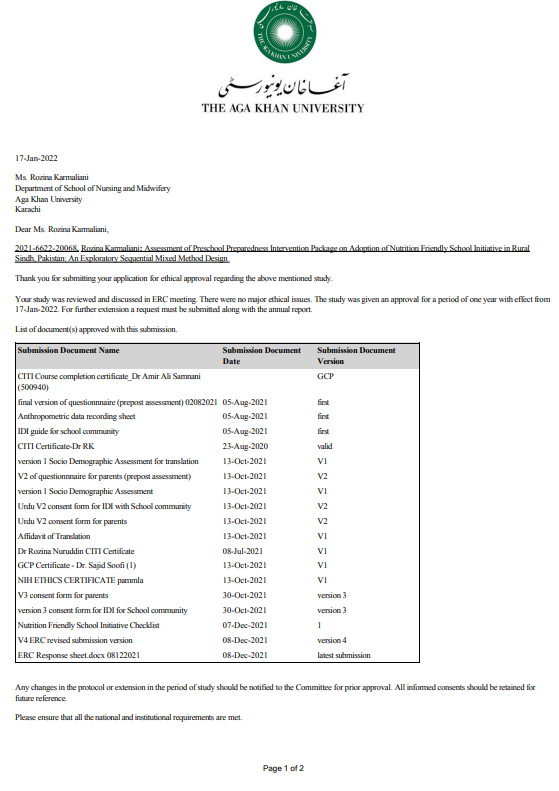
**

**
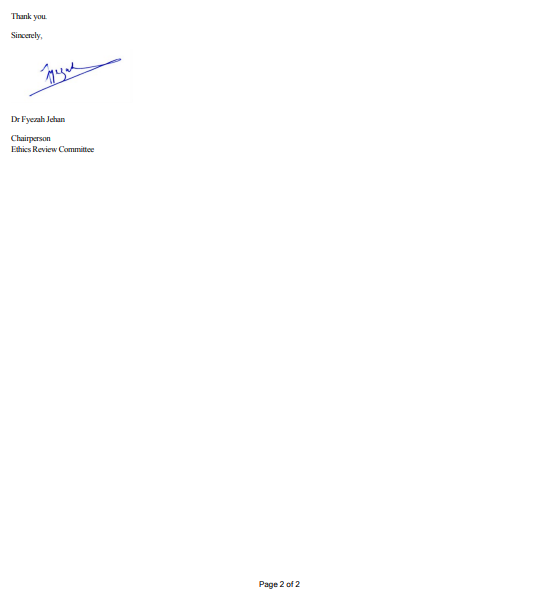
**

**Annexure 4: Inform consent form for Parents / Caregivers**

**Title of study:** Assessment of Preschool Preparedness Intervention Package on Adoption of Nutrition-Friendly School Initiative in Rural Sindh, Pakistan: An Exploratory Sequential Mixed Method Design

**Principal Investigator:** Dr Amir Ali Samnani

**Committee members:** Dr. Rozina Karmaliani, Dr. Rozina Nuruddin, Dr. Sajid Soofi, Dr. Pammla Petruka

**Introduction:** Schools provide the most effective and efficient way to reach young children at influential stages in their lives when lifelong nutritional patterns are formed. Furthermore, schools may provide a setting to introduce nutrition information and technologies to the community and can lead the community in advocating for policies and services that promote good nutrition. Since you are eligible to participate in this study, I would like to invite you to join this research study.

**Background:** The burden of all forms of malnutrition is significantly high in Pakistan. Malnutrition refers to deficiencies, excesses, or imbalances in a person’s intake of energy. According to Pakistan National Nutrition Survey (NNS-2018), the prevalence of stunting (short height for age) is 40%, whereas, wasting among children 6-59 months is on the rise from 8.6% in 1997 to 15.1% in 2011 and 17.7% in 2018 [3]. This is the highest rate of wasting in Pakistan’s history since 1965. [3]. Besides this, over 40% of disadvantaged children under 5 years globally have neurodevelopmental problems resulting in deficits in social, emotional, and educational functioning into adulthood [4]. Early childhood development (ECD) interventions delivered to children aged less than 5 years have been clearly shown to have substantial and sustained impacts on long-term cognition and neurodevelopmental outcomes

**Purpose of the Study:** The purpose of this study is to pilot the implementation of Nutrition Friendly School Initiatives (NFSI) in preschool settings and to assess its outcomes in three major schools of AKESP, in Karachi, Pakistan by engaging school community (preschool teachers, ECD experts, Principal and representative from Parent teacher committees, etc.) Parents and preschool children, in adopting a nutrition-friendly school initiative through an iterative process.

**Procedure:** You are being requested to participate in this study, whereby you will be assessed twice (pre & post-training) with some basic Knowledge-related questions regarding health and nutrition. You will be provided with training on the Health and Nutrition manual to impart knowledge and develop an understanding of the importance of early years of life, malnutrition and its consequences, food and nutrition, Health and hygiene, child safety, protection, and a safe environment. Furthermore, a Nutrition assessment (using Mid Upper Arm Circumference (MUAC), weight, Height, and edema) of your child/ren will also be carried out within school premises to assess the nutrition status of the child/ren twice

**Possible Risks or Discomforts:** There are no major risks associated with the study, however, if participants feel hesitant in responding to any question further clarity will be provided and they have an opportunity to skip any particular question to prevent any anxiety or stress. If needed, the interview will be interrupted till the participant feels comfortable.

**Benefits:** There are numerous benefits linked with participation in this study, Firstly, participants will get a chance to attend the training on nutrition and health promoting curriculum. Secondly, this pool of trained staff will receive a letter/certificate of appreciation from the section head of the school after the successful completion of this study. Moreover, any child identified with any sign of malnutrition or health condition will be referred to a health facility for further care or need-based counseling.

**Financial Consideration:** No financial compensation is involved for your participation in this study. However, participants will be served with refreshment during training sessions.

**Voluntary Participation:** Enrolment in this study is completely voluntary. You will have full right to withdraw from the study at any time and you may refuse to answer some or all questions if you do not feel comfortable. Refusal to participate will not in any way affect your child’s enrollment or studies in school.

**Confidentiality:** The information shared by you will be kept confidential. Any identity-revealing information will not be mentioned in the questionnaire. All the data will be kept under a locked cabinet and the electronic files will be password protected. Access to study materials will be limited to the principal investigator and research team. The findings of the study will be shared or published in a journal without revealing your identity.

**The available source of Information**

If you have any queries or questions, you may contact the primary researcher: Dr. Amir Ali Samnani, Aga Khan University, Pakistan.

**Agreement to Participate:** I have read and understood this consent form, and I agree to participate in this study and allow for the following

1. Anthropometric assessment of my child/ren.
2. To participate in training and for pre-post assessment.

I understand that there are no major risks or benefits attached to this study. I also understand that the information that I provide, will be kept confidential. I voluntarily choose to participate, but I understand that my consent does not take away any legal rights. I further understand that nothing in this consent form is intended to replace my applicable federal, state, or local laws. I understand that a copy of this form will be provided to me.

Participant’s Name and Signature: ___________ Student ID: _________Date: ___________

Principal investigator’s Signature: _______________________ Date: ______________
